# Comparison of videofluoroscopy and impedance planimetry for the evaluation of oesophageal stenosis: a retrospective study

**DOI:** 10.1007/s00330-016-4516-y

**Published:** 2016-08-23

**Authors:** Martina Scharitzer, Johannes Lenglinger, Wolfgang Schima, Michael Weber, Claudia Ringhofer, Peter Pokieser

**Affiliations:** 1 Department of Biomedical Imaging and Image-guided Therapy, Medical University of Vienna, Waehringer Guertel 18-20, 1090 Vienna, Austria; 2 Department of Visceral Surgery and Medicine, University Hospital Bern, Bern, Switzerland; 3 Department of Surgery, Medical University of Vienna, Vienna, Austria; 4 Department of Diagnostic and Interventional Radiology, KH Goettlicher Heiland, KH der Barmherzigen Schwestern, St. Josef-KH, Vienna, Austria; 5 Unified Patient Division, Teaching Center, Medical University of Vienna, Vienna, Austria

**Keywords:** Oesophageal stenosis, Deglutition disorders, Cineradiography, Impedance planimetry, Tablets

## Abstract

**Objectives:**

To compare videofluoroscopy that included a tablet test with impedance planimetry (EndoFLIP^®^) for the evaluation of oesophageal stenosis in patients with dysphagia.

**Methods:**

In 56 patients, videofluoroscopic examinations following the transit of a 14-mm tablet were retrospectively reviewed and correlated with impedance planimetry findings, a catheter-based method using impedance planimetry to display the oesophageal diameter estimates. Additional findings assessed were the occurrence of symptoms during tablet passage and evaluation of oesophageal motility.

**Results:**

Impaction of the tablet occurred in 31/56 patients; nine showed a moderate delay (2–15 s), three a short delay (<2 s) and 13 no delay of tablet passage. Both methods showed a significant correlation between tablet impaction and oesophageal diameter <15.1 mm, as measured by impedance planimetry (*p* = 0.035). The feeling of the tablet getting stuck was reported by seven patients, six showing impaction of the tablet (four with an EndoFLIP-diameter < 13 mm, two with a diameter of 13–19 mm) and one showing delayed passage (EndoFLIP diameter of 17 mm).

**Conclusions:**

Videofluoroscopy and impedance planimetry correlate significantly regarding tablet impaction and residual oesophageal lumen. A standardized 14-mm tablet is helpful in demonstrating oesophageal strictures in dysphagic patients. Triggering of subjective symptoms provides valuable information during a videofluoroscopic study.

***Key Points*:**

*• A 14-mm tablet can demonstrate oesophagogastric junction narrowing in patients with dysphagia.*

*• Type of passage of a tablet enables estimation of oesophageal luminal diameter.*

*• Videofluoroscopy and impedance planimetry correlate significantly regarding tablet impaction and residual oesophageal lumen.*

## Introduction

Dysphagia is a commonly encountered clinical symptom and includes various morphological and functional aetiologies. It may affect swallowing of solids, liquid consistencies, tablets alone or any combination of these [1]. Swallowing studies have proven to be a radiological modality of choice allowing a significant number of relevant diagnoses [2]. Within the last decade, a change in videofluoroscopic findings, with an enlarged spectrum of benign oesophageal stenosis-like rings, webs and small-calibre oesophaguses, has been observed [3, 4]. Eosinophilic oesophagitis, a relatively recently recognized disorder, is now one of the leading causes of oesophageal dysphagia among adults [4, 5]. Recognition of oesophageal narrowing is mandatory for treating and alleviating dysphagia in these patients. The diagnosis of oesophageal narrowing is still problematic, since endoscopy and static single-spot radiography may fail to achieve optimal maximal distension and evaluation of residual lumen diameter [6]. Gentile et al. described endoscopic detection rates of oesophageal narrowing of 15 % in patients with eosinophilic esophagitis when compared to radiological evaluation reported on barium oesophagrams [7].

Videofluoroscopy is considered the method of choice for the visualization of bolus flow related to structural movement along the upper digestive tract from mouth to stomach in real time [8–10]. In addition to the detection of structural and functional abnormalities, the effects of different bolus volumes, bolus consistencies and compensatory manoeuvres have also been observed. In patients with a history of obstruction during swallowing, and particularly the presence of solid food dysphagia, including a tablet test in the videofluoroscopic examination protocol may allow additional important information about bolus transport and the level of delay of bolus passage [11].

Impedance planimetry (EndoFLIP^®^) is a novel diagnostic technique for the evaluation of measurements of cross-sectional areas in the alimentary tract and has been assessed in several sphincter regions of the gastrointestinal tract including the upper oesophageal sphincter, the oesophagogastric junction and the anorectal region [12]. One clinical application is the assessment of oesophageal mechanical properties in vivo. By filling an intra-oesophageal bag containing an array of electrodes, the reaction of the oesophageal wall to varying distension volumes can be assessed by measuring cross-sectional areas, while simultaneously evaluating intrabag pressure and a distensibility index that combines both results. Impedance planimetry creates a real-time geometric image of the area along up to 16 points at each electrode pair. An estimate of the balloon diameter is provided from the measured cross-sectional area at the centre of each electrode pair. Indications for use of impedance planimetry in dysphagic patients with impaired oesophageal bolus transport and preserved mucosal integrity have been suggested [13, 14]. However, distinct indications for impedance planimetry in the clinical setting have not been established as yet. In contrast to lower oesophageal sphincter (LES) pressure assessed by high resolution manometry, oesophagogastric (EGJ) distensibility correlates with oesophageal emptying and clinical response. Therefore, it is a better parameter to evaluate efficacy of treatment for achalasia [15]. The purpose of this study was to compare videofluoroscopy including a tablet test and impedance planimetry for the evaluation of oesophageal stenosis in adult patients with dysphagia.

## Materials and methods

### Patient population

**Fig. 1:**
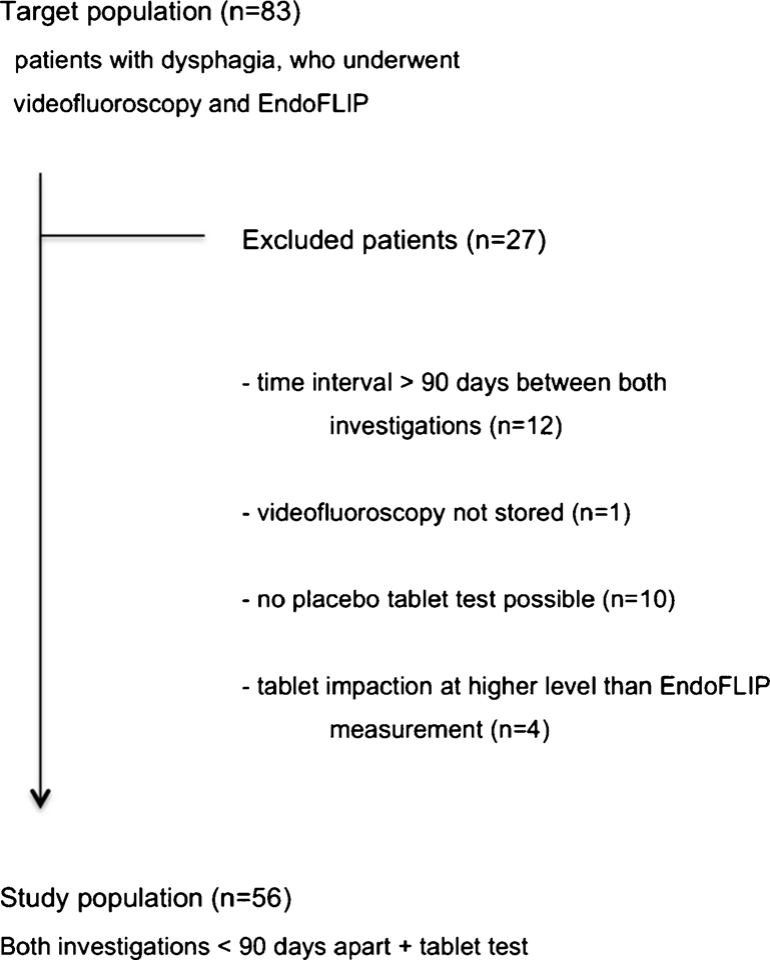
Flowchart of patient enrolment

The study was designed as a retrospective study with the aim of comparing videofluoroscopy that included a tablet test and impedance planimetry for the evaluation of oesophagogastric junction in patients with dysphagia. The protocol for this study was reviewed and approved by the institutional review board of our institution. A search of radiology and impedance planimetry files between September 2010 and February 2015 revealed 83 patients who underwent both investigations. Twelve of these patients were excluded from the study because the time interval between both investigations exceeded 90 days, and one videofluoroscopic study was not stored on videotape because of technical problems. In 10 patients, no tablet test was included in the protocol. In four patients, the tablet impaction occurred at a higher level than the EndoFLIP measurement was performed and therefore no direct comparison between both measurements was possible (Fig. 1). Patients were referred to our institution on the basis of their clinical symptoms of solid food dysphagia and suspicion of oesophageal narrowing and the endoscopic lack of an underlying diagnosis.

### Videofluoroscopy

Patients were investigated in the upright and prone position on a remotely controlled fluoroscopy unit (Diagnost 55, Philips, Eindhoven, Netherlands). A drink of barium suspension (250 % g/v; Prontobario HD, Gerot, Vienna, Austria) was recorded on videotape during passage through the pharynx and oesophagus in standing right and left oblique positions, respectively. Afterward, single swallows in the prone position were recorded, followed by two to four double-contrast images after the ingestion of a packet of effervescent agent and immediate gulping of a barium suspension, displaying the oesophagus and the oesophagogastric junction. Placebo tablets of a spherical shape, measuring 14 mm in diameter, and consisting of sugar to ensure quick dissolving if there was a risk of impaction, were used (patients with diabetes were informed about the sugar content of 0.1 BE per tablet). These tablets were administered only after evaluating the passage of a thin-liquid barium suspension to exclude pharyngeal dysfunction with misdirected swallowing and risk of aspiration. In the upright position, the patients were asked not to chew the tablet and to keep it in their mouth together with 10–15 ml of thin-liquid barium suspension, and to swallow immediately on the examiner’s cue. Transit of the tablet was followed videofluoroscopically. After primary tablet retention, the patient was requested to take one or two additional swallows of the barium suspension. If no tablet passage into the stomach was observed after 3 min, the investigation was terminated.

### Impedance planimetry

Impedance planimetry was performed using the commercially available Endo-FLIP^®^ system (Endoluminal Functional Lumen Imaging Probes, Crospon Ltd, Galway, Ireland). The catheter (EF 325, Crospon Ltd, Galway, Ireland) was fitted with a cylindrical bag, distensible to a diameter of 25 mm with nearly infinite compliance. Inside the bag the catheter was equipped with an array of 17 voltage-sensing electrodes at 5-mm distances over an 8-cm length. An alternating current was applied by excitatory electrodes proximally and distally to the measuring electrodes. The bag was filled with a proprietary electrolyte solution acting as electrical conductor between the electrodes. Additionally intrabag pressure was measured by an inbuilt solid-state pressure transducer. Impedance measurements between pairs of electrodes at 10-Hz frequency were converted to real-time images of adjacent cross-sectional areas. Combining impedance readings at the smallest cross-sectional area with pressure measurements allowed EGJ distensibility to be calculated. The catheter was inserted into the stomach transnasally after topical anaesthesia. Intragastric placement was assured by distending the bag to a 50-ml filling volume with the bag assuming a cylindrical shape, typically with an increase in intrabag pressure of less than 30 mmHg. Then the bag was emptied to 2 ml residual volume and the pressure transducer was zeroed to intragastric pressure. With a filling volume of 10–15 ml the catheter was retracted until it was centred at the level of the manometrically determined lower oesophageal sphincter and the bag displayed an hourglass shape. Stepwise bag distensions with volumes of 20, 30, 40 and 50 ml were performed. EGJ distensibility was dynamically measured over at least 30 s at each volume excluding swallows. At each volume patients were asked to swallow once and to refrain from swallowing for the following 60 s. Times of swallows were protocolled. The 30-s distension measurements commenced 30 s after a swallow, assuming that lower oesophageal sphincter pressure had returned to resting state at this point of time. In case this 30-s period was interrupted by a swallow, coughing or gagging the procedure was repeated. After completion of the measurement, the bag was deflated and the catheter withdrawn. Data were stored on a USB drive for analysis on a PC. Distensibility parameters represent 30-s median values at the level of the narrowest cross-sectional area. For correlation with videofluoroscopy, EGJ opening diameter and a distensibility index (square millimetres per millimetre of Hg) at the maximal filling volume of 50 ml were used. All impedance planimetry procedures were conducted and analysed by two investigators (J.L., C.R.).

### Image evaluation:

Two radiologists (P.P., M.S.), both with more than 10 years’ experience in videofluoroscopy, independently reviewed the videofluoroscopic studies to classify tablet passage through the oesophagus. They were informed that all patients were referred with symptoms of dysphagia, but were not provided with any other information about the patients.

If the tablet passed through the oesophagus without delay, the study was considered normal. According to previously published data about the transit time of a capsule in healthy volunteers [16], oesophageal transit was categorized as follows: a short tablet delay lasting less than 2 s was registered as normal; a retention with the tablet remaining in the same position for 2–15 s was considered a moderate delay and more than 15 s was considered a severe delay. If the tablet did not move for 3 min, it was characterized as impaction. Patients’ subjective perceptions of a solid bolus delay during swallowing were registered. In addition to the time of tablet delay, the anatomical site of tablet retention was recorded. Additional abnormal oesophageal motility disorders were evaluated and scored as none, moderate or severe motility disorders. Scored findings of tablet impaction were correlated with the values from impedance planimetry on the basis of the description regarding the location of the lesion as a coordinate.

### Statistical analysis

Data were analysed using a statistical software program IBM SPSS Statistics version 22.0 (IBM Corp, Armonk, NY). The Fisher–Halton–Freeman test was used to compare tablet results for different oesophageal diameters as well as tablet impaction for different gradings of oesophageal motility disorders. A *p* value of 0.05 or less was considered to indicate a statistically significant result. *k* statistics were used to assess interobserver agreement: *k* values of 0.61–0.80 were indicative of substantial agreement, and *k* values of 0.81–1.0 were indicative of almost-perfect agreement. Repeated measures analysis of variance was performed for comparison of diameter during different filling volumes of the impedance planimetry bag. An a priori power analysis was based on a chi^2^ test to compare the two groups (<15.1 mm vs. ≥15.1 mm). This test revealed that 48 patients were needed to obtain a power of 80 % (alpha 5 %, two-sided) to detect a medium effect (epsilon = 0.5).

## Results

We included 56 patients who underwent videofluoroscopy and impedance planimetry between September 2010 and February 2015. The mean time interval between the two studies was 37 days (range 1–90 days). The patients received no symptom-based therapy within this interval. In this final study group (40 men, 16 women), the mean age was 50.2 years (range 18–83 years). All patients suffered from solid food dysphagia, 22 of them (39.3 %) from bolus impactions. Medical history revealed that 13 patients had undergone fundoplication, six patients had had myotomy of the oesophagogastric junction, five dilation of the EGJ due to achalasia, one patient suffered from achalasia without previous intervention and six patients had histologically proven eosinophilic oesophagitis. Among 48 of 56 patients (in eight patients non-local endoscopy reports were not available), 31 (64.6 %) had no visible stenosis at the oesophagogastric junction at endoscopy, 13 (27.1 %) had a stenosis and four (8.3 %) had signs of eosinophilic oesophagitis without evident stenosis.

**Fig. 2:**
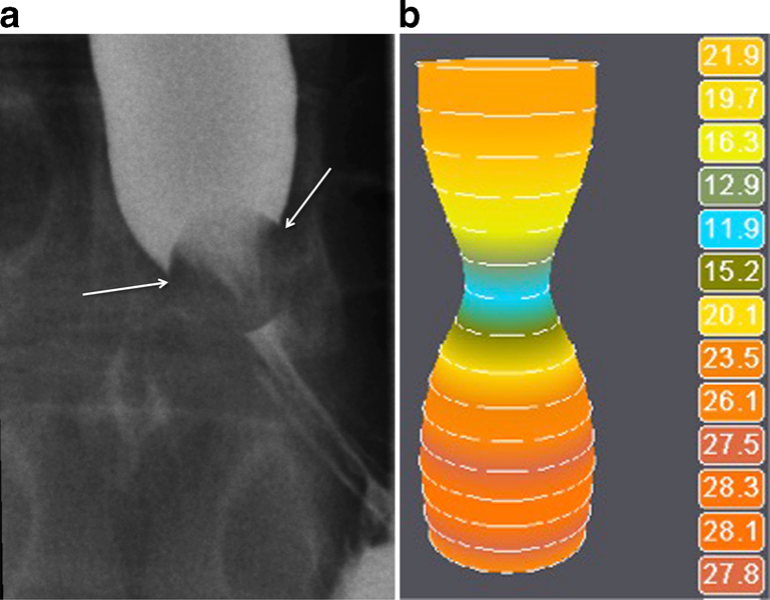
A 19-year-old patient with dysphagia and achalasia. **a** Impaction of the tablet at videofluoroscopy for >3 min. **b** EndoFLIP lumen at 50 ml shows a minimal diameter of 12.4 mm (*blue*)

**Fig. 3:**
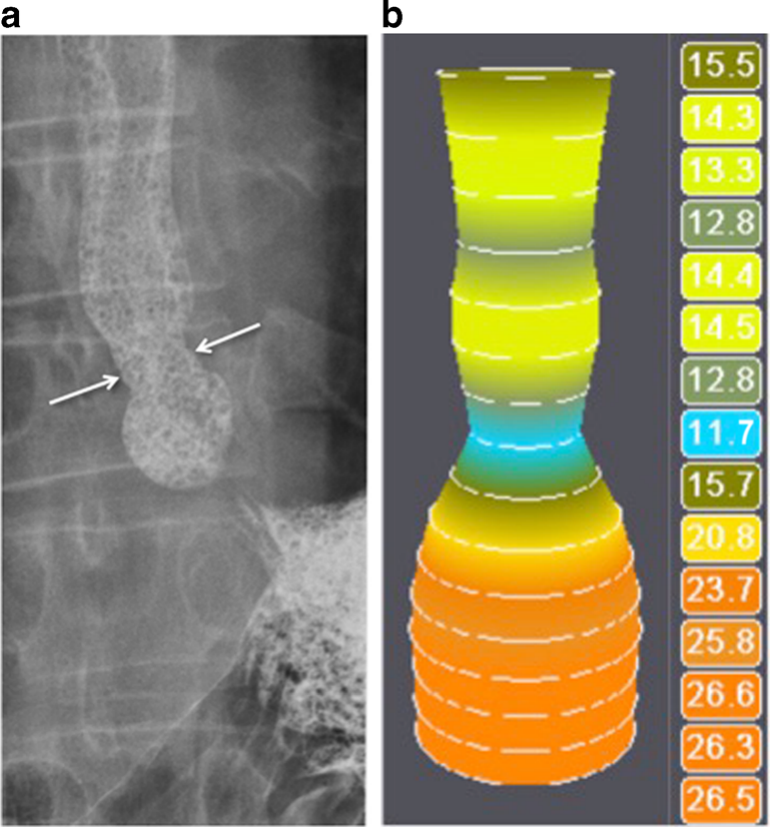
Peptic stenosis in a 37-year-old patient with gastroesophageal reflux and dysphagia. **a** The tablet was impacted at the level of the narrowing (*arrows*). **b** EndoFLIP lumen at the oesophagogastric junction is 11.7 mm (*blue*) and 12.8 mm (*dark green*) 2 cm more proximal, corresponding to the videofluoroscopically detected stenosis

**Fig. 4:**
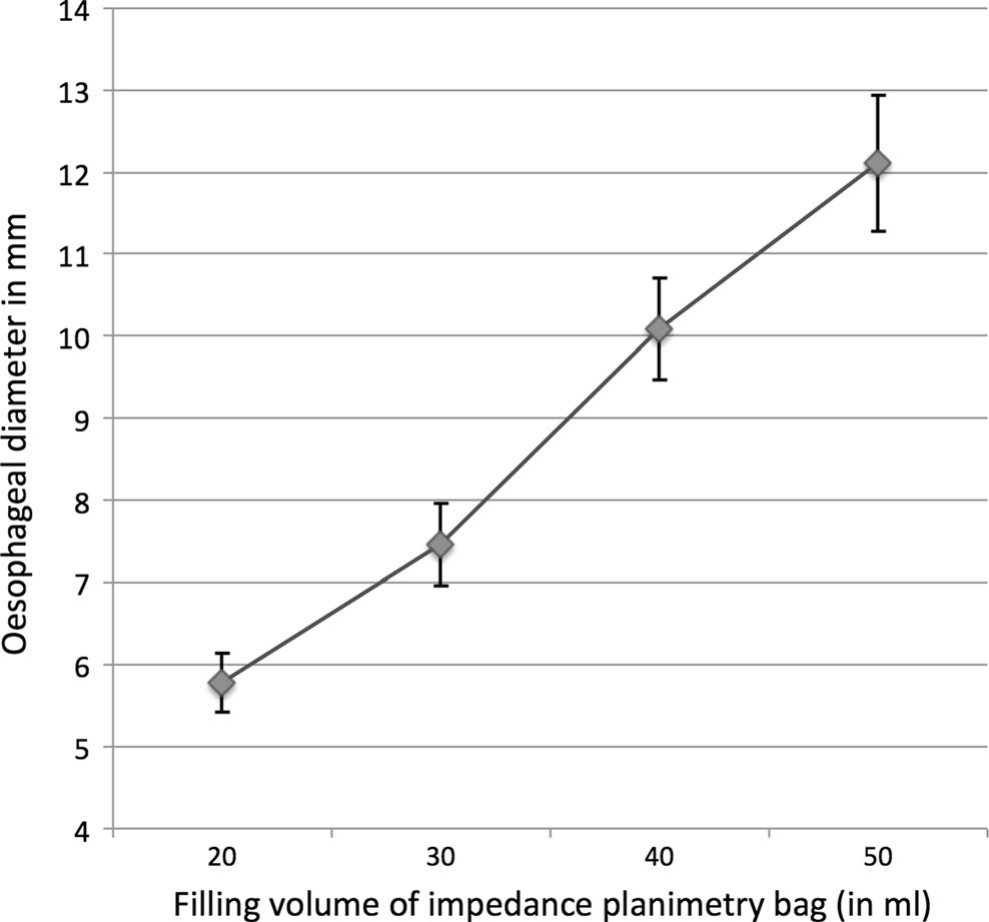
Average oesophageal diameter (mm) for different filling volume of impedance planimetry bag (ml), *error bars* represent 95 % confidence intervals

In 13 (23.2 %) patients, the tablet passed into the stomach without any delay, in three patients there was a localized delay up to 15 s, in nine (16.1 %) patients the tablet passage along the oesophagogastric junction took up to 3 min and in 31 (55.4 %) patients the tablet was impacted (Figs. 2 and 3). When comparing tablet impaction and a delay of more than 15 s for an EndoFLIP oesophageal of diameter of less than 15.1 mm, a significantly statistical correlation was found (*p* = 0.035; Table 1). When comparing tablet impaction and a delay of greater than 15 s for an EndoFLIP oesophageal diameter of less than 14.1 mm, results were not statistically significant (*p* = 0.136). Raters showed perfect agreement for the assessment of tablet passage (*k* = 1.0). Comparing observations of relevant, not relevant or questionable stenosis by single- and double-contrast oesophagography between the readers, *k* values were 0.918 for single-contrast studies and 0.958 for double-contrast studies. The observations of relevant, not relevant and uncertain stenosis assessed by videofluoroscopy and compared to EndoFLIP diameter are shown in Table 2. Comparing findings on single- and double-contrast radiography with regard to impaction of the tablet, statistical correlation was significant for both readers (*p* < 0.001). In eight (14.3 %) patients, one or both readers found no relevant stenosis on single- or double-contrast radiography, or were not sure about the relevance of a stenotic finding, but the tablet test showed impaction. In these eight patients, EndoFLIP revealed an endoluminal diameter between 6.8 and 14.5 mm (mean 11.6 mm). Symptoms during impaction occurred in 7/54 patients (two records regarding triggering of symptoms were missing from the files): 6/30 patients with impaction and 1/9 patient with a delay of greater than 15 s had the feeling of the tablet getting stuck. Of the six symptomatic patients with impaction, four had an EndoFLIP diameter of less than 13 mm, two had a diameter of less than 18 mm and one symptomatic patient with delayed passage had an EndoFLIP diameter of 17 mm. No symptoms were reported during tablet passage by the patients who had a minimal or no delay. In our study group, sensitivity and specificity of impedance planimetry for less than 15.1 mm versus at least 15.1 mm diameter were 79.1 % and 53.8 %, respectively. Stepwise distension of the EndoFLIP bag showed linear increase in oesophageal diameter (Fig. 4).

**Table 1:** Placebo tablet transit correlated with oesophageal diameter as measured by impedance planimetry

		Oesophageal tablet transit		Total	*p* value*
		Impaction or localized delay > 15 s	Short delay ≤ 15 s or normal passage		
Impedance planimetry diameter	≥15.1 mm	6 (46.2 %)	7 (53.8 %)	13 (100 %)	0.035
	<15.1 mm	34 (79.1 %)	9 (20.9 %)	43 (100 %)	
Total		40 (71.4 %)	16 (28.6 %)	56 (100 %)	

*p values evaluated by Fisher–Halton–Freeman test

**Table 2:** Findings of single- and double-contrast radiography correlated to impedance planimetry diameter

Impedance planimetry diameter	Reader 1							Reader 2						
	Single-contrast			Double-contrast			*p* value	Single-contrast			Double-contrast			*p* value*
	Stenosis	No stenosis	Uncertain	Stenosis	No stenosis	Uncertain	0.577	Stenosis	No Stenosis	Uncertain	Stenosis	No stenosis	Uncertain	0.470
<15.1 mm	30	9	4	31	10	2		32	9	2	32	10	1	
≥15.1 mm	9	4	0	8	5	0		9	4	0	8	5	0	
Total	39	13	4	39	15	2		41	13	2	40	15	1	

*p values evaluated by Fisher-Halton Freeman test

When assessing the correlation between no, mild or severe oesophageal motility disorders and tablet impaction, no significant correlation was found (*p* = 0.104 for reader 1 and *p* = 0.098 for reader 2). Kappa values for both readers were 0.728. There were no complications during the passage of the tablet.

## Discussion

Oesophageal narrowing due to benign or malignant abnormalities is usually manifested by symptoms of dysphagia if the residual lumen is reduced to 50 % of its regular diameter or less than 13 mm [17]. Although continuous drinking of contrast media and the use of a double-contrast technique provide good distension of the oesophagus, objective radiological measurement of oesophageal lumen patency still remains a challenge. The additional use of a barium-based tablet or a bread sphere as an aid in the detection of occult oesophageal stenosis was described for the first time in 1956 and was used for several decades [18–20]. Since that time, various solid food tests with radiopaque tablets, marshmallows, solid food or even magnetic disc tablets have been used to evaluate the level and the remaining dimension of oesophageal narrowing [21–26]. An ingested, intact tablet with a defined diameter, when swallowed as a whole, provides a gauge by which to evaluate the true size of the residual oesophageal lumen diameter if the tablet stops or its passage is delayed above an oesophageal stenosis. The size of 13 mm was originally chosen owing to the concordance to endoscopes with an outer diameter of 36 F [27], and this proved to correlate strongly with the presence of dysphagia in patients with oesophageal ring-like stenoses of less than 13 mm [17]. The retention of a tablet may also occur temporarily in healthy persons without dysphagia, as shown by Chisaka et al. [16]. Several factors influence the tablet passage, including size, shape and consistency of the tablet, age and position of the patient, and volume of water ingested [28, 29]. Nevertheless, tablet passage that requires more than 15–20 s in the upright position may suggest a true structural abnormality of the oesophagus [16, 24].

Impedance planimetry enables real-time visualization of the mechanical properties of the oesophageal lumen and measurement of oesophageal diameter for given distension volumes. As an invasive technique, it requires expertise and is used in specialized tertiary referral centres only. Studies have already described the use of EndoFLIP in patients with achalasia [30] and in oesophageal stenosis due to eosinophilic oesophagitis [31–33]. EndoFLIP has shown a poor correlation between endoscopic and EndoFLIP estimates of oesophageal and oesophagogastric distensibility, possibly because distending pressure during endoscopy is variable, estimation is qualitative and the circumstances under endoscopy are non-physiologic [32]. EndoFLIP has also been shown to be a better predictor of food impaction in patients with eosinophilic oesophagitis than endoscopy findings and mucosal eosinophil count [33]. Rohof et al. showed a better correlation between EndoFLIP and a timed barium oesophagogram than manometry for predicting clinical outcome in the management of patients with achalasia [15]. This can be explained by the difference between distensibility and contractility of the lower oesophageal sphincter (LES). The EndoFLIP catheter has been used as “smart bougie” to gauge EGJ diameter during fundoplication [34] and peroral endoscopic myotomy [14, 35]. Recently it was shown that final intraoperative EGJ cross-sectional area inversely correlates with postoperative dysphagia assessed with the Eckardt score and determines gastroesophageal reflux in achalasia patients after peroral endoscopic myotomy or Heller myotomy [36, 37]. In contrast to manometry, swallowing of a tablet also helps to evaluate the distensibility that is responsible for the development of solid food dysphagia. To our knowledge, a comparison between videofluoroscopy using a tablet test and impedance planimetry has not been published as yet. Our results showed a significant correlation between impaction of the tablet and an oesophageal diameter of less than 15.1 mm on impedance planimetry. Comparison between tablet impaction and oesophageal diameter of less than 14.1 mm showed a smaller difference in tablet impaction probability as a result of a high probability of tablet impaction in patients with EndoFLIP diameter between 14 and 15 mm (*n* = 7). Although the size of the tablet used was 14 mm and therefore a bit smaller than the threshold diameter of less than 15.1 mm by impedance planimetry, this may be due to the fact that filling of the intra-oesophageal bag implies active distension compared to passive impaction of the tablet. In one patient, tablet passage was delayed for less than 15 s, and EndoFLIP values were less than 15.1 mm; in two patients, tablet passage was normal and EndoFLIP values were abnormal. This may be because larger distension volumes may elicit intermittent secondary or tertiary contractions with higher contractile tone in the oesophageal musculature [31].

When the evaluations of relevant oesophageal stenosis during single- and double-contrast radiography were compared, there were minor differences between both readers. Correlation with EndoFLIP was significantly better with the tablet test, and both readers showed identical results with the latter test, indicating a high inter-reader reliability owing to the simplicity of evaluation of this test. Another radiological study showed similar results, since the use of a marshmallow bolus improved the detection of mucosal rings and was inversely related to ring calibre [21]. In our study, in eight patients, single- and double-contrast radiography showed no relevant stenosis or allowed no definitive diagnosis, but impaction of the tablet, as well as impedance planimetry, revealed a significant narrowing. Previous studies have shown better results for the full-column technique than for double-contrast techniques in the depiction of oesophageal stenosis [6, 38]. Nevertheless, endoscopic correlation was either not part of these studies or showed lower sensitivity rates than radiological evaluation when it was included in the study.

Correlation with the subjective perception of bolus delay of the tablet while swallowing reveals valuable key information in the radiologic evaluation, and it has been shown to occur more often in patients with oesophageal dysmotility than with narrowing [9]. Nevertheless, almost one-third of patients with oesophageal stenosis and consecutive bolus transport delay localize their symptoms in the neck [39]. Therefore, oesophageal assessment that includes a solid bolus should be considered in all patients with dysphagia, particularly when pharyngeal findings are inconsistent with the clinical history [9]. Manometric investigations have shown differences in flow resistance and levels of muscle tension during the transition phases in subjects who report the perception of solid bolus dysphagia [40]. Observations of a relationship between subjective and objective measures of oesophageal solid bolus clearance differ significantly with either marked correlation or no correlation with the perception of retention in patients who report solid food dysphagia [41–43]. In our study, all patients with subjective symptoms had impaction of the tablet. Evaluation of oesophageal motility in patients with solid food dysphagia and tablet impaction showed no significant correlation between the two findings. However, patients with tablet impaction above the gastroesophageal junction were not included in the study. In general, the clinical utility of precise distinction among abnormal oesophageal motility and type of dysphagia seems to be limited [44].

The limitations of our study include the fact that the tablet test was not possible in all patients who were referred for solid food dysphagia. An intact oropharyngeal phase of swallowing with proof of absence of pharyngeal paresis or aspiration below the vocal cords is a precondition for a solid bolus test. In addition, in four patients, tablet impaction occurred at a level higher than the location of the EndoFLIP measurement and direct comparison was therefore not possible. Nevertheless, a tablet may reveal bolus-specific oesophageal dysmotility at any level of the oesophageal body, which may be the underlying cause for inhibited bolus passage and solid food dysphagia in symptomatic patients. The selection of the patient population could have biased our study results as a result of its retrospective design. Patients with endoscopically diagnosed severe stenosis are likely underrepresented, because these patients were not referred for EndoFLIP. However, the study population comprised patients with solid food dysphagia with or without history of impaction, which could not be explained endoscopically. Another limitation is that the assessment of a precise lumen diameter of less than 14 mm and greater than 15 mm is not possible with the videofluoroscopic tablet test. However, this size threshold has been shown to reliably separate between symptomatic and non-symptomatic oesophageal stenoses [17].

## Conclusion

Videofluoroscopy and impedance planimetry use distinct evaluation methods and measurement parameters, but correlate significantly regarding tablet impaction and residual oesophageal lumen. A standardized 14-mm placebo tablet is helpful for demonstrating oesophageal strictures in dysphagic patients and for estimating residual lumen size, as well as provoking typical symptoms during impaction. Therefore, a tablet test should be included in every patient with solid food dysphagia during the videofluoroscopic work-up to confirm or exclude a clinically significant narrowing of the oesophageal lumen.
